# Dynamic Weight Agnostic Neural Networks and Medical Microwave Radiometry (MWR) for Breast Cancer Diagnostics

**DOI:** 10.3390/diagnostics12092037

**Published:** 2022-08-23

**Authors:** Jolen Li, Christoforos Galazis, Larion Popov, Lev Ovchinnikov, Tatyana Kharybina, Sergey Vesnin, Alexander Losev, Igor Goryanin

**Affiliations:** 1 School of Informatics, University of Edinburgh, Edinburgh EH8 9YL, UK; 2 Department of Computing, Imperial College London, London SW7 2BX, UK; 3 Faculty of Mathematics and Information Technology, Volgograd State University, 400062 Volgograd, Russia; 4 Medical Microwave Radiometry (MMWR) Ltd., Edinburgh EH10 5LZ, UK; 5 Pushchino, Library for Natural Sciences of the Russian Academy of Sciences, 119991 Moscow, Russia; 6 Biological Systems Unit, Okinawa Institute of Science and Technology, Onna 904-0495, Japan

**Keywords:** breast cancer, passive microwave radiometry (MWR), network architecture search (NAS), weight agnostic neural network (WANN), CMA-ES algorithm

## Abstract

Background and Objective: Medical microwave radiometry (MWR) is used to capture the thermal properties of internal tissues and has usages in breast cancer detection. Our goal in this paper is to improve classification performance and investigate automated neural architecture search methods. Methods: We investigated extending the weight agnostic neural network by optimizing the weights using the bi-population covariance matrix adaptation evolution strategy (BIPOP-CMA-ES) once the topology was found. We evaluated and compared the model based on the F1 score, accuracy, precision, recall, and the number of connections. Results: The experiments were conducted on a dataset of 4912 patients, classified as low or high risk for breast cancer. The weight agnostic BIPOP-CMA-ES model achieved the best average performance. It obtained an F1-score of 0.933, accuracy of 0.932, precision of 0.929, recall of 0.942, and 163 connections. Conclusions: The results of the model are an indication of the promising potential of MWR utilizing a neural network-based diagnostic tool for cancer detection. By separating the tasks of topology search and weight training, we can improve the overall performance.

## 1. Introduction

Medical microwave radiometry (MWR) is used to obtain the internal tissue temperature of the body [1]. This is done by measuring the naturally emitted thermal radiation from the tissues. Additionally, it is a noninvasive, nonionizing, and cost-effective approach. Due to the device’s accuracy, there are already multiple clinical applications using the temperature readings and patterns to identify various conditions [1,2,3,4,5,6,7,8,9,10,11]. In this paper, we focus on using MWR to detect breast cancer. This is viable since the growth rate of tumors is correlated with the tissues’ temperature [12,13]. In addition to the thermal information of the tissue, we can derive from MWR the cancer cells’ reproduction rate and mutagenesis risk levels [14].

MWR is a relatively new clinical imaging technique. Thus, for it to be adopted successfully, an artificial intelligence (AI) diagnostic tool needs to be developed in parallel. The diagnostic tool alleviates the need for training clinicians to interpret the data and prevents workload increase, while also providing a more accurate prediction. Thus, our first objective is to further improve the diagnostic accuracy of the model. Furthermore, while this research is focused on breast cancer, MWR has clinical applications at different anatomical locations [1,14] and in various conditions. To reduce the development time of models for each of these cases, we explore adapting automatic machine learning (AutoML) techniques for MWR data.

There has been previous work with AutoML for MWR using a cascade correlation neural network (CCNN) [15]. Subsequent improvements were made by expanding the pool of layers and activation functions the model could explore [15,16]. Despite the improvements, it was not able to outperform predefined architectures [16]. However, it resulted in a small network that was desirable when considering edge computing and hardware limitations. Various classification models have been explored in the past, such as deep neural networks, convolution neural networks, support vector machines, and random forests [15,16]. Additionally, a rule-based classification model was introduced that improved the interpretability of the results [16].

In summary, our contributions in the field of MWR for breast cancer detection are two-fold. First, we evaluate the application of weight agnostic Neural Network (WANN) [17] on MWR data and compare it against the CCNN that was used in previous research [15]. Secondly, we improve the WANN model for MWR classification. Once the topology of the network is found using WANN, we use the bi-population covariance matrix adaptation evolution strategy (BIPOP-CMA-ES) [18] to find the optimal weight candidates. Combining the WANN and BIPOP-CMA-ES strategies, we obtain state-of-the-art classification performance on MWR breast cancer data. Furthermore, we conclude that a random search strategy to optimize the weights yields better results than those achieved by a gradient descent method for architectures generated by WANNs for MWR data.

## 2. Methods

### 2.1. Cascade Correlation Neural Network

Cascade correlation neural network (CCNN) is an early neural architecture search (NAS) technique for supervised tasks [19]. The idea of a CCNN is to start with a minimum-sized network, input and output layers, and add one additional node at a time until convergence. The steps of the algorithm are as follows [19]:

1. Initialize network topology with input and output nodes.
2. Create a pool of candidate hidden layer nodes initialized at different starting weights. The hidden layer node takes input from all previous layers. Its output is connected to the output layer nodes. Each candidate node is trained until convergence.
3. From the pool of candidates, select and add to the network the candidate node that maximizes the magnitude of the correlation between the output and target on the validation set. The input weights of the added hidden layer nodes are frozen.
4. If the correlation does not improve or improves by a small margin, then terminate the algorithm. Otherwise, proceed to step two.

**Figure 1:**
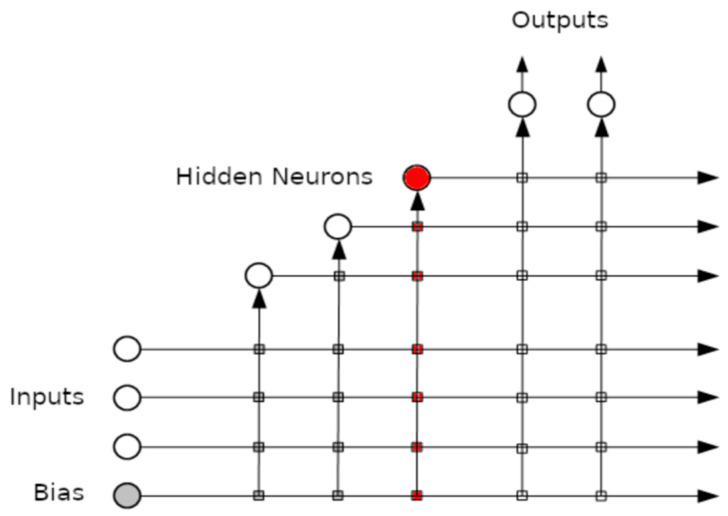
The cascade correlation neural network after adding three hidden layers.

An example of the connections created after adding the 3rd hidden layer can be seen in Figure 1.

The loss function we used is the cross-correlation loss and optimize using stochastic gradient descent. The optimizer’s learning rate was set to $5\times 10^{-6}$. Each of the nodes used a sigmoid activation function. Furthermore, their weights were sampled from a Gaussian distribution with a mean of 0 and 0.5 standard deviation, and the bias was set to 0. A combination of batch normalization and dropout layers (rate of 0.5) was added after the node. The candidate node pool size was set to 30. Additionally, we reinitialized the weights of the output layers after each iteration to avoid being stuck in a bad local minimum.

### 2.2. Weight Agnostic Neural Network

Another NAS method is the weight agnostic neural network (WANN) approach [17]. The main difference between WANN and CCNN is that during the architecture search, the weights of the model are not trained. Instead, a set of fixed shared weights are used to evaluate the average performance. According to the authors, the idea behind this is to automatically find architectures that have inductive biases and can perform well in their given task without training.

Inspired by genetic evolution, WANN starts with a population of small initial networks. The initial networks consist of the input and output layers. However, the nodes between the layers are sparsely connected. Then, once the population of networks is established, a series of fixed shared-value weights, which we set to [−2, −1.5, −1, −0.5, +0.5, +1, +1.5, +2], are used to evaluate the performance of each topology. The evaluation metric we used is the geometric mean between the predicted and actual values.

The topologies were ranked on the basis of three criteria: mean performance across all fixed shared weights, best performance between any of the fixed shared weights, and the number of connections. Similar to the authors of WANN, we used the Non-dominated Sorting Genetic Algorithm II (NSGA-II) [20] to sort the network topologies based on the previous criteria. NSGA-II is a multi-objective sorting genetic algorithm that combines elitism and does not require a priori selection of shared parameters. The highest-ranking topologies were selected for the next step using the tournament algorithm [21].

Once the new population was selected, they were subsequently varied to generate the next generation of population. There were three mutation operations used to increase the complexity of the model. First, a node can be inserted between two connected nodes. Secondly, a new connection can be added between two existing nodes. Finally, the activation function of a node can be changed according to the list in Table 1. This process of evaluating, ranking, and generating a new population was repeated until there was no longer improvement.

The hyperparameters for the WANN model are summarized in Table 2. These hyperparameters were defined through extensive experimentation. Specifically, we searched 200 generations, each having a population size of 200. The existence of the initial connections between input and output layers was set to 0.2. For topology variation, we set a likelihood of changing the activation function to 0.5, adding a node to 0.25, and creating a new connection to 0.25. Finally, for the tournament algorithm, we set the size to 4.

### 2.3. Weight Agnostic Neural Network BIPOP-CMA-ES

For MWR breast cancer data, we can determine patterns and relationships between the points to identify high-risk patients. By using a WANN model, the resulting architecture relies on creating node connections to identify similar properties, in addition to new ones. Thus, the architecture acts as a prior. However, there are more subtle cases to distinguish between those that are low- and high-risk. Specifically, these cases will be when the tumors’ growth rate slows down. This can be achieved through weight optimization once the optimal architecture has been found.

Based on the research results of WANN, while it performs better than chance in most cases, it is not able to outperform fixed topologies that have had their parameters tuned [17]. A way to circumvent this is by taking the best topology found by the WANN model and proceeding to optimize the parameters via a gradient descent algorithm. However, a network with various activation functions results in a difficult gradient traversal [17].

**Figure 2:**
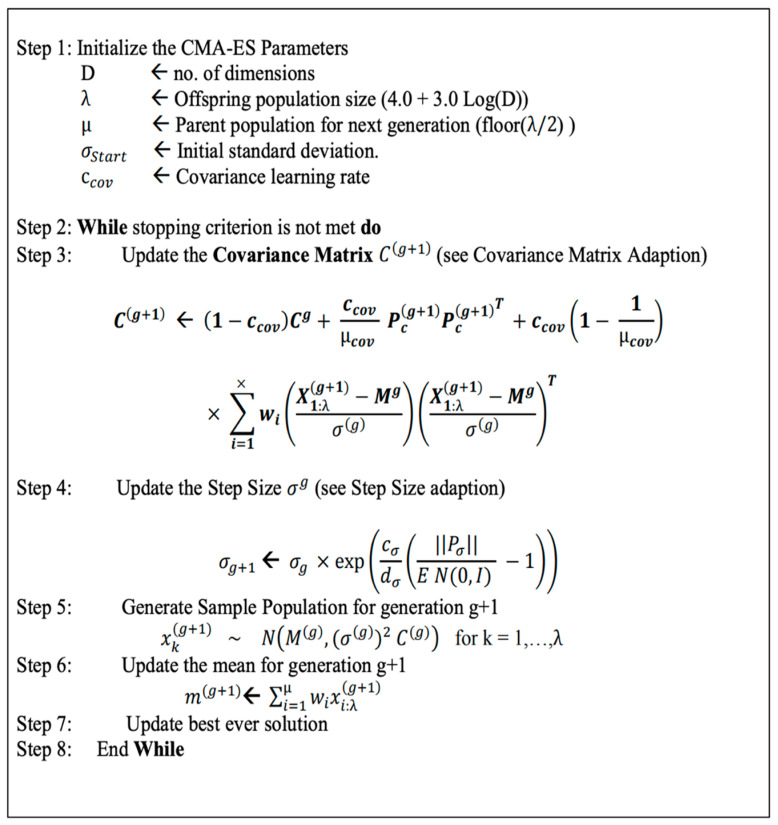
The pseudo-code of the CMA-ES algorithm [23].

Thus, a better way of optimizing the weights is through a black-box optimization method such as the CMA-ES algorithm [17,22]. With the randomized search of the CMA-ES, it is suitable for a rigged landscape in which there are many bad local minima, discontinuities, and noise. The steps of the CMA-ES algorithm are summarized in Figure 2.

We used a variant of the CMA-ES algorithm, the bi-population covariance matrix adaptation evolution strategy (BIPOP-CMA-ES) [18]. Through our experiments, we found it to perform better than CMA-ES. BIPOP-CMA-ES uses a variable population size. It initially starts with a small population size, which we set to 50, and doubles after each restart. Additionally, to speed up convergence, we fine-tuned the initial single shared weight of the model. We achieved this by linearly evaluating values between −2 and 2. Finally, the cross-entropy loss was used to find the best fit.

## 3. Results

### 3.1. Data

The MWR breast cancer data were captured using the MMWR-2020 (RTM-01-RES) (http://www.mmwr.co.uk accessed on 26 July 2022) device in clinics over the world. The data were classified as either low or high risk for breast cancer. Classification of the patients was done by clinicians using MWR, mammography, and/or biopsy data as necessary. In total, 4912 cases were recorded, with 4377 as low-risk and 535 as high-risk. Subsequently, we class-balanced and separated our data into train, validate, and test sets using 60%, 20%, and 20%, respectively. The data are publicly available from http://www.mmwr.co.uk/dataset_clean breast.csv accessed on 26 July 2022.

**Figure 3:**
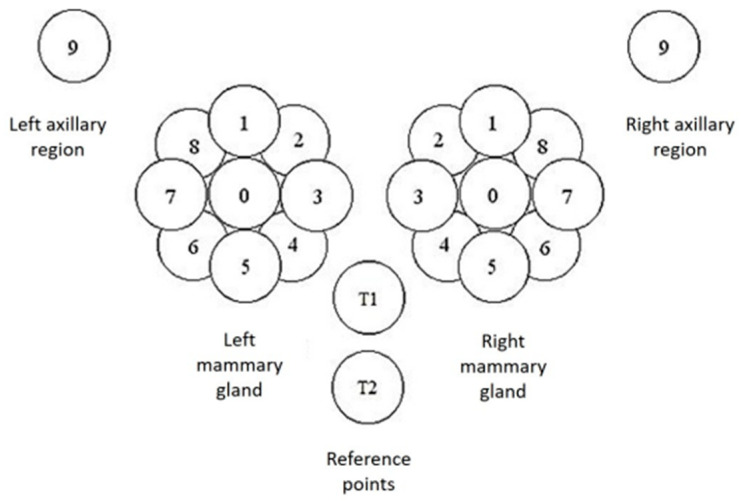
Capture positions for each mammary gland (0–8) and at the axillary point (9). There are the additional T1 and T2 reference points under the chest. Each position was captured on the skin and at a depth of 3–5 cm.

The MWR data consist of temperature readings of the skin surface and internally at a depth of 3–5 cm. There were in total 44 points recorded on the mammary glands and surrounding regions. On each gland, a point was recorded on the nipple and nine equidistant around the nipple. Additionally, two reference points were captured just below the chest. Finally, two more points were captured under each axillary area. Each of these points was captured both on the skin and at a depth. The positions described are shown in detail in Figure 3.

### 3.2. Experimental Results

Each model was trained three times using a different random number generator seed. For the analysis, we obtained from the test set results the mean and standard deviation. We explored additional NAS techniques that are available from the Neural Network Intelligence [24] package. Specifically, the exploration strategies we investigated are Differentiable Architecture Search (DARTS) [25,26], regularized evolution [27], and Tree-structured Parzen Estimator (TPE) [28]. The model search space is based on a previously developed fully connected (FC) network [15], with the extended search space specified in Table 3. To also consider the impact of our model, we compared the output results of our model against the other models using a statistical significance t-test. We consider a *p*-value of <0.05 to be statistically significant.

**Figure 4:**
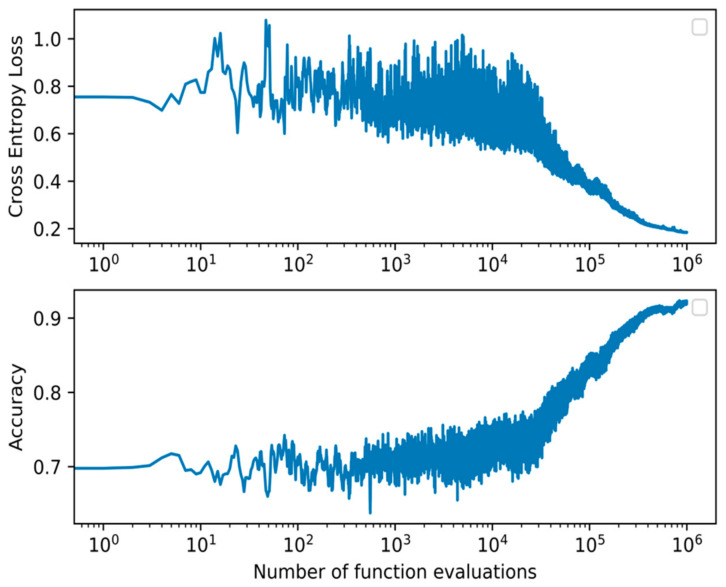
Cross-entropy loss and accuracy curve of the BIPOP-CMA-ES optimization algorithm during training of the best WANN topology.

From the models presented, the best performing on the test set across all metrics is the proposed WANN BIPOP-CMA-ES. The model obtained an average F1-score of 0.933, accuracy of 0.932, precision of 0.929, recall of 0.942, and required 163 connections. It needed $10^{6}$ function evaluations before converging on the validation set. When assessing the *p*-value, our model’s results are statistically significant compared to all other models. All but the WANN model result in a much lower *p*-value of at most 0.005. The WANN model is still statistically significant but with a marginal value of 0.045. The summary of the results is shown in Table 4. The accuracy and loss during training of the WANN BIPOP-CMA-ES can be seen in Figure 4.

To further analyze the models, we also compared the mean element-wise difference of the values between WANN BIPOP-CMA-ES against WANN and FC-Evolution (second-best-performing model). They obtained respectively a mean difference of 0.49 (±0.57) and 0.76 (±0.78). Furthermore, we observed from the networks’ output that most erroneous cases from the WANN were close to the threshold of value 0.5. On the other hand, the erroneous cases of the FC models differed by a large amount. In contrast to WANN’s *p*-value, it was the worst-performing model when no weight optimizations were done.

**Figure 5:**
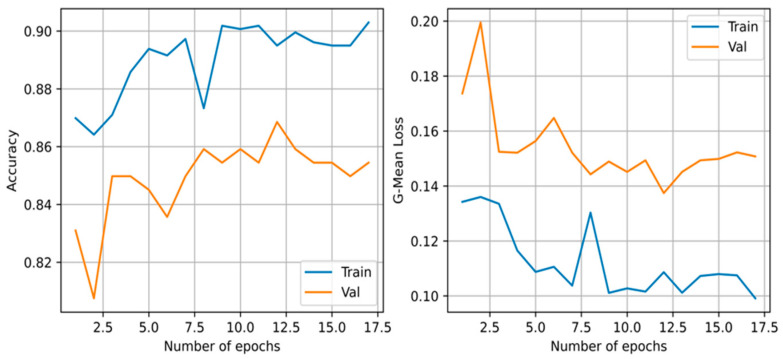
The geometric mean loss and accuracy of the training and validation set of the cascade correlation neural network during training.

The architecture and optimized weights obtained from the CCNN had an average performance on the F1-score with a value of 0.809. It required a total of 17 iterations before terminating due to the validation loss not decreasing given patience of five iterations for this execution. The accuracy and loss during training can be viewed in Figure 5. However, it required a total of 672 connections before converging.

Additionally, we evaluated the robustness of the WANN’s generated topology to weight changes. Specifically, we evaluated the performance of the network when using fixed, randomly generated, and fine-tuned shared weights. For each of these cases, we ran them 10 times and took their average. In Table 5, we show a summary of the results of the test set. By tuning the weights, we improved the performance. In contrast, using random weights, we obtained the worst performance.

## 4. Discussion

Similar to the observations of the authors of WANN [17], the generated model we obtained can achieve better performance than that of chance based on the generated topology of the network. We can further improve the performance by optimizing the weights. However, despite the improvement when we used a gradient descent optimizer, the performance is still subpar to that of the other models. We gained a much larger improvement in performance when utilizing a random search evolution strategy.

Our proposed model obtains the best performance on all metrics evaluated and has the least number of connections. The WANN and CCNN models have a small number of connections as they start from a minimum-sized network and gradually expand. In contrast, FC-Evolution, FC-TPE, and FC-DARTS search from a predefined architecture space that allows them to start from a large or small network. The trend of these approaches was to opt for larger network sizes early in their architecture search, as they have a higher learning capacity. Additionally, we showed that the predicted results from the network are statistically significant when paired against all other evaluated models. However, while the WANN’s performance is the worst, it has the highest *p*-value. This is probably an indication of the importance of the architecture and that inductive biases are maintained to some degree, despite weight training.

A general summary of the advantages and disadvantages of all models is shown in Table 6. Furthermore, there are domain-specific advantages of WANN BIPOP-CMA-ES and an extension of NAS for healthcare applications. First, the generated topology of the network is optimized to have a small number of parameters and sparse connections due to the inclusion of the model size as a minimization objective [17]. This allows the model to be deployed on low-end devices and on already existing clinical hardware, which is particularly important for accessibility to low- and mid-income countries. Second, we decrease the development time, as architecture tunning through manual trial-and-error is reduced. Additionally, the model becomes more accessible to nontechnical experts, such as clinicians, as they do not require vast knowledge of machine learning to develop a model. Without this barrier, they can more effectively contribute to and improve the diagnostic tool. Finally, from the aforementioned benefits, it reduces the complexity and time of adapting MWR for different anatomical locations and pathologies.

The mutation operations of WANN only increase the complexity of the network topology. For future work, we will expand it to include operations such as deleting nodes and deleting connections so there is more flexibility in defining the architecture. In addition, there is no crossover mutation operation in the WANN model, which will reduce population diversity. Hence, we will explore different crossover mutation operations [29,30,31] and restart techniques to increase model performance [32,33]. Finally, we will investigate cross-trial information sharing, such as including in the mutation pool more complicated building blocks generated from previous trials.

Furthermore, we will look at improving our model by searching for the best loss function [34], utilizing a one-shot learning search [35,36], and conducting a hyperparameter search [37]. We will also compare against additional NAS methods such as reinforcement learning-based searching methods [38,39], ensemble methods [40], and transfer learning [41]. Our main aim is to determine if a performance improvement can be made without significantly increasing computational complexity.

## Figures and Tables

**Table 1:** Summary of the pool of activation functions that the weight agnostic neural network samples from.

Name	Equation	Range
Linear	(1) f(x)=x	(−∞,∞)
Binary step	(2) f(x)={1 for x≥0 0 for x<0}	{0, 1}
Sin	(3) f(x)=sin(x)	[−1, 1]
Cosine	(4) f(x)=cos(x)	[−1, 1]
Sigmoid	(5) $f\left(x\right)=\frac{1}{1+e^{-x}}$	(0, 1)
Gaussian	(6) $f\left(x\right)=e^{-x^{2}}$	(0,1]
TanH	(7) f(x)=tanh(x)	(−1,1)
Inverse	(8) f(x)=−x	(−∞,∞)
Absolute Value	(9) f(x)=abs(x)	[0,∞)
ReLu	(10) f(x)={x for x≥0 0 for x<0}	[0,∞)
Squared	(11) $f\left(x\right)=x^{2}$	[0,∞)

**Table 2:** The hyperparameter selection for the weight agnostic neural network.

Hyperparameter	Value
Generations	200
Population Size	200
Change Activation Probability (%)	50
Add Node Probability (%)	25
Add Connection Probability (%)	25
Initial Active Connections (%)	20
Tournament Size	4

**Table 3:** The model search space of a fully connected neural network. It was used by DARTS, regularized evolution, and TPE NAS algorithms.

Model Search Space	Range
Number of Layers	[2, 8]
Number of Units per Dense Layer	{32, 64, 128, 256, 512}
Activation Function	See Table 1
Batch Normalization	{Include, not Include}
Dropout Rate	{0, 0.1, 0.25, 0.5}
Skip Connection	{Include, not Include}

**Table 4:** The summary of the results on the test set for all models. Each model was trained three times, in which a different random number generator seed was used each time, and the metrics presented are the average and standard deviation of those runs.

Model	F1-Score	Accuracy	Precision	Recall	Connections	*p*-Value
FC-Evolution	0.905 ± 0.004	0.903 ± 0.004	0.892 ± 0.012	0.919 ± 0.009	1342 k ± 373 k	<<0.05
FC-TPE	0.9 ± 0.018	0.9 ± 0.017	0.903 ± 0.016	0.897 ± 0.02	1254 k ± 124 k	<<0.05
FC-DARTS	0.849 ± 0.037	0.846 ± 0.04	0.834 ± 0.047	0.866 ± 0027	1166 k ± 124 k	<<0.05
CCNN	0.809 ± 0.011	0.816 ± 0.011	0.825 ± 0.012	0.795 ± 0.033	672 ± 28	<<0.05
WANN	0.673 ± 0.013	0.697 ± 0014	0.727 ± 0.042	0.631 ± 0.047	163 ± 9	<0.05
WANN BIPOP-CMA-ES	0.933 ± 0.007	0.932 ± 0.008	0.929 ± 0.005	0.942 ± 0.021	163 ± 9	-

**Table 5:** Performance of WANN on the test set using different weight schemes.

WANN	F1-Score	Accuracy	Precision	Recall
Random weight	0.5209	0.5591	0.5571	0.4892
Shared weight	0.5979	0.6628	0.7212	0.5105
Tuned shared weight	0.6546	0.6947	0.7363	0.5892

**Table 6:** Summary of the main advantages and disadvantages of the models explored.

Model	Advantages	Disadvantages
FC-Evolution	With an evolutionary algorithm, it is simpler to define population mutations. Evolution strategies can be parallelized easier.	The diversity of the connections is limited to predefined layers. Has a fixed outer structure, such as the number of layers or units. Favors larger model sizes.
FC-TPE	It is a sequential model-based optimization method that approximates the architecture’s hyperparameters based on previous results. Architecture search is computationally efficient.	The diversity of the connections is limited to predefined layers. Has a fixed outer structure, such as the number of layers or units. Favors larger model sizes.
FC-DARTS	Gradient descent is used to search for optimal architecture hyperparameters. Since it is a one-shot NAS approach, it can converge to an optimal architecture and weights faster. To achieve this, it uses two-step training: one to search for model architecture and the other for parameter optimization.	Training data are divided into architecture and weight training. For a small (pathological) dataset like the one used, it limits the performance as the divided data do not share the same distribution. The diversity of the connections is limited to predefined layers. Has a fixed outer structure, such as the number of layers or units. Favors larger model sizes.
CCNN	Small network size. Freezes previously added nodes to improve training time.	Slow to train as each node in the pool has to be trained individually. Depends on a larger node pool size to avoid bad local minima. Training individual nodes at a time can make the model more prone to overfitting. Node connections are predefined. Thus, there is limited diversity and complexity in the resulting architecture.
WANN	Small network size (model size is an optimization objective). Evolution strategies can be parallelized easier. Promotes connection sparsity between the nodes. No weight optimization leads to faster architecture convergence. Allows for diverse and complex connections between nodes that are not practical to define manually. Finds a more optimal network architecture compared to other methods explored. The performance is exclusively obtained from the network’s architecture (which has inductive biases).	Subpar performance when compared to other models. The model’s weights are not optimized for the given task and hence they are underutilized. The WANN architecture search space is unconstrained. Thus, there are no clear stopping criteria. Too few trials might result in a poor-performing network, or too many can lead to unnecessary high compute time.
WANN BIPOP-CMA-ES	Small network size (model size is an optimization objective). Evolution strategies can be parallelized easier. Promotes connection sparsity between the nodes. Allows for diverse and complex connections between nodes that are not practical to define manually. By separating the architecture search and weight training, it benefits from the inductive biases from the WANN and weights fine-tuning from the BIPOP-CMA-ES to improve performance. A small architecture during the first phase results in fast weight training during the second phase.	Low performance when using the more common gradient descent methods used in neural network training. It is currently unclear which WANN model should be selected to be trained. The highest-performing WANN on a given set of metrics might not yield the best performance when weight has been optimized. While it is computationally efficient to train a single WANN, it is computationally demanding when training multiple WANN models.

## Data Availability

The data are publicly available from http://www.mmwr.co.uk/dataset_clean breast.csv accessed on 26 July 2022.
